# Supplementary material for: 1H-Nuclear Magnetic Resonance Analysis of Urine as Diagnostic Tool for Organic Acidemias and Aminoacidopathies
Source: Metabolites. 2021 Dec 20;11(12):891. doi: 10.3390/metabo11120891 (PMC8704601; doi:10.3390/metabo11120891)
Supplement: Supplementary file 1 [file metabolites-11-00891-s001.zip › metabolites-1482661-supplementary.pdf]

[1]H-NUCLEAR MAGNETIC RESONANCE ANALYSIS OF URINE AS DIAGNOSTIC TOOL FOR ORGANIC ACIDEMIAS AND AMINOACIDOPATHIES

Ninna Pulido[1,2], Johana M. Guevara-Morales[2], Alexander Rodriguez-López[3,4], Álvaro Pulido[5], Jhon Díaz[4], Ru Angelie Edrada-Ebel[6*], Olga Y. Echeverri-Peña[2*]

ADDITIONAL FILES

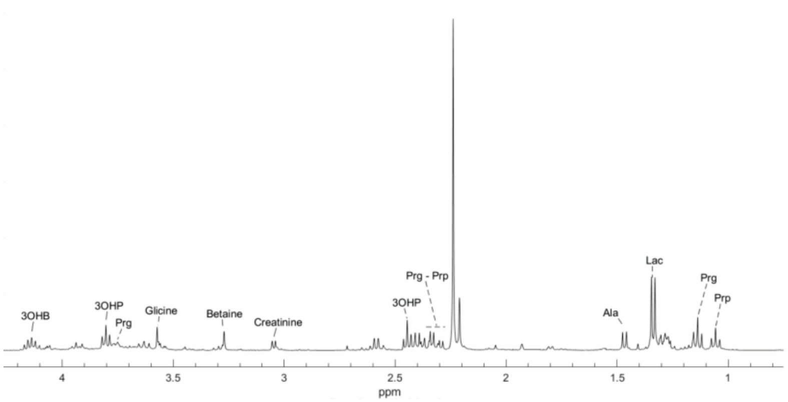

Figure S1 *Propionic Acidemia*: ^{1}H-NMR spectrum. Signals for propionylglycine (Prg), 3-hydroxy-propionic acid (3OHP) and propionic acid (Prp).

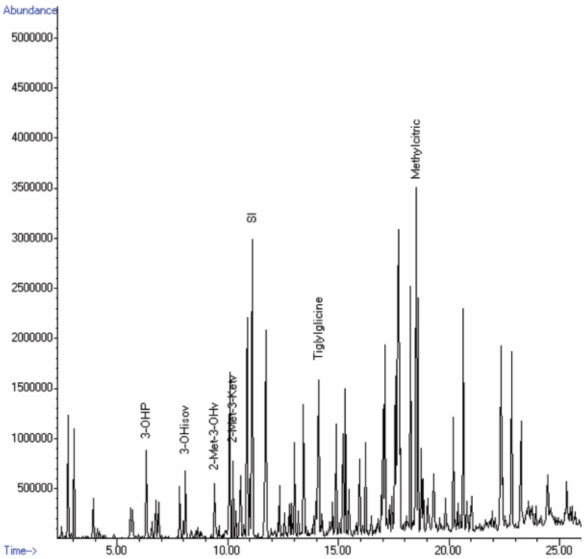

Figure S2: *Propionic Acidemia:* Chromatogram obtained by GC-MS. Signals for 3-Hydroxy-propionic acid (3-OHP), 3-Hydroxy-isovaleric acid (3-OHisov), 2-Methyl-3-Hydroxy-valeric (2-Met-3-OHv), 2-Methyl-3-Ketovaleric acid (2-Met-3-Ketv), Tyglylglycine and Methylcitric.

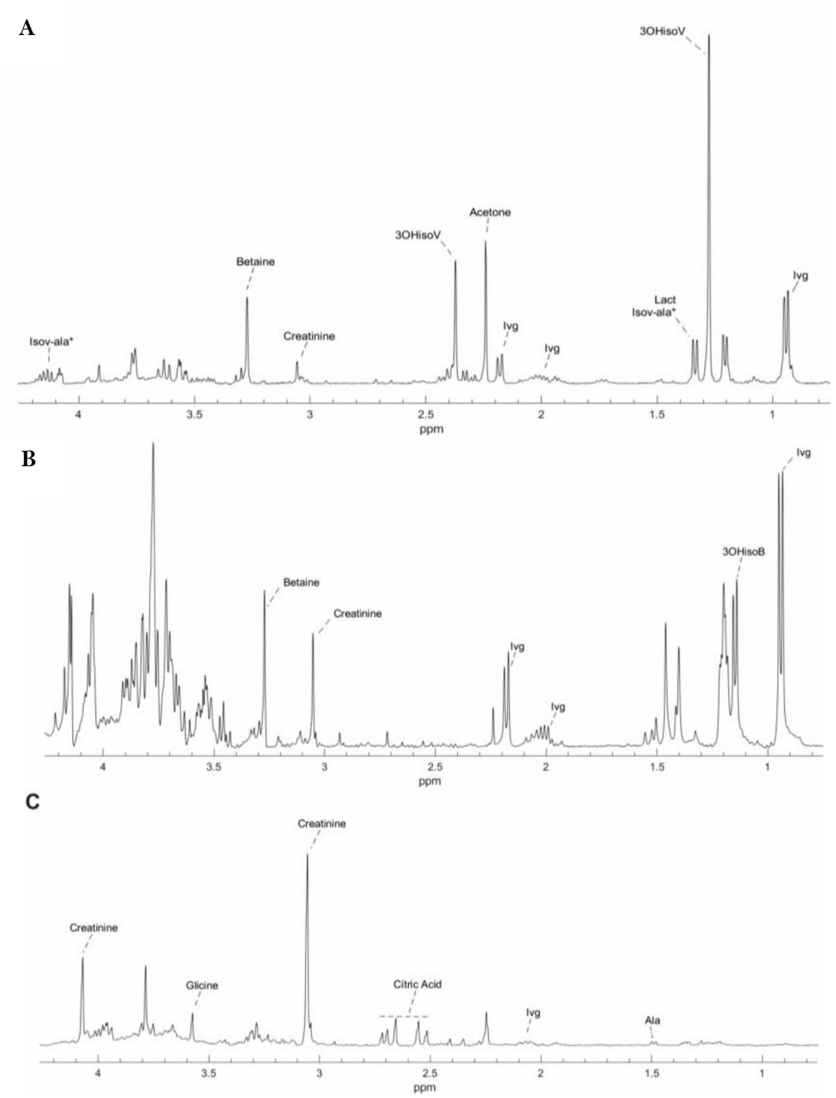

Figure S3. *Isovaleric Acidemia*: ^{1}H-NMR spectrum. **(a)** Isovaleric Aciduria, signals for isovalerylglycine (Ivg), 3-hydroxyisovaleric acid (3-OHisoV) and isovalerylalanine (Isov-ala), (**b)** in acute crisis signals for 3-Hydroxybutyric acid (3-OHB), 3-Hydroxy-isovaleric acid (3-OH-isov) and Isovalerylglycine, and **(c)** follow up with signals similar normal ^{1}H-NMR spectrum.

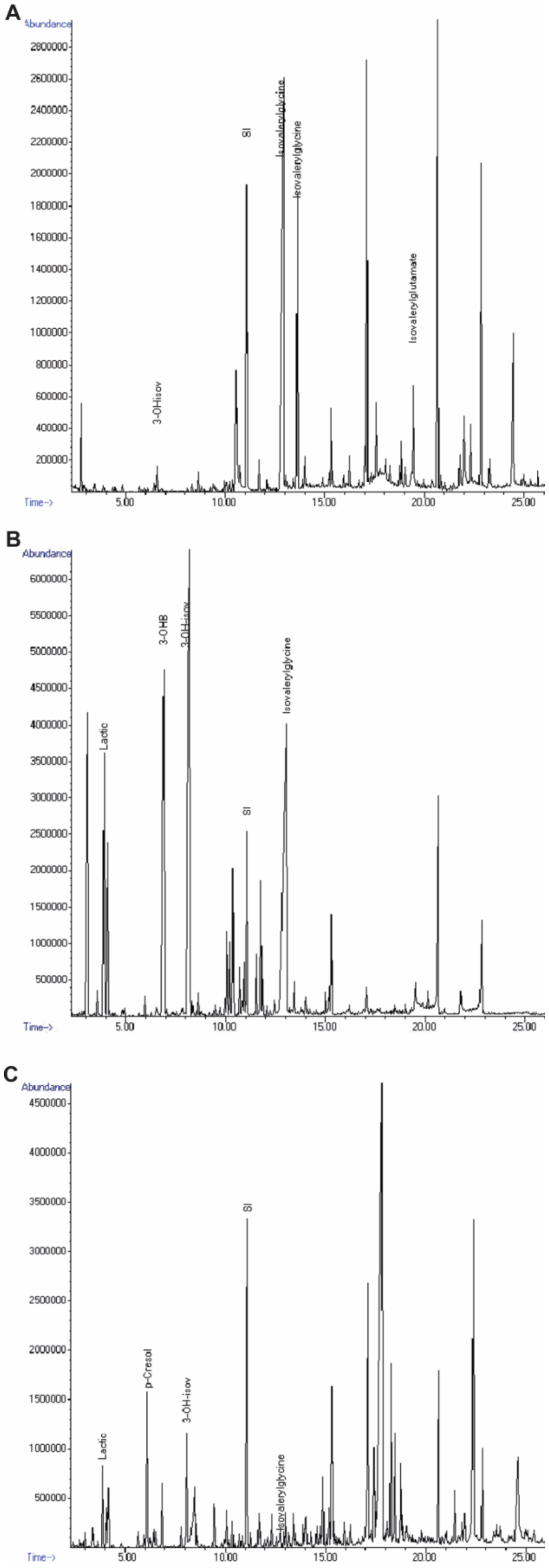
A
Abundance
Time-->
3-OH-isov
SI
Isovalerylglycine
Isovalerylglycine
Isovalerylglutamate
B
Abundance
Time-->
Lactic
3-OHB
3-OH-isov
SI
Isovalerylglycine
C
Abundance
Time-->
Lactic
p-Cresol
3-OH-isov
SI
Isovalerylglycine

Figure S4. *Isovaleric Acidemia:* Chromatograms obtained by GC-MS. **(a)** Isovaleric Aciduria, signals for Isovalerylglycine and Isovalerylglutamate **(b)** in acute crisis signals for Lactic acid, 3-Hydroxybutyric acid (3-OHB), 3-Hydroxy-isovaleric acid (3-OH-isov) and Isovalerylglycine, and **(c)** follow up with signals for lactic acid, 3-Hydroxy-isovaleric acid (3-OH-isov) and Isovalerylglycine.

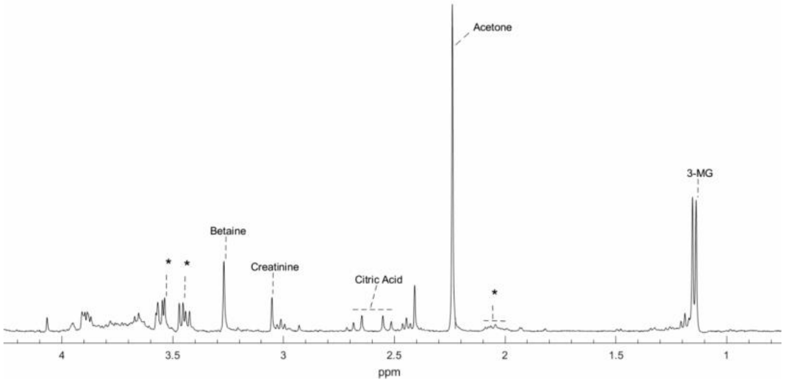

Figure S5. *3-Methylglutaconic Acidemia:* ^{1}H-NMR spectrum. signals for 3-methylglutaric acid (3-MG). The presence of very weak signals with no defined multiplicity was also found between 1.97 and 2.09 ppm along with doublet pairs at 3.45 and 3.54 ppm, that could correspond to the methylated protons of 3-methylglutaconic acid (*)

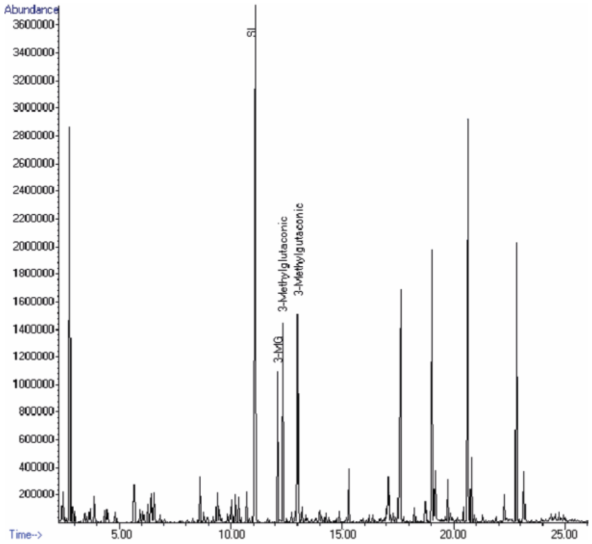

Figure S6. *3-Methylglutaconic Acidemia:* Chromatogram obtained by GC-MS. Signals for 3-Methylglutaric acid (3-MG) and 3-Methylglutaconic acid.

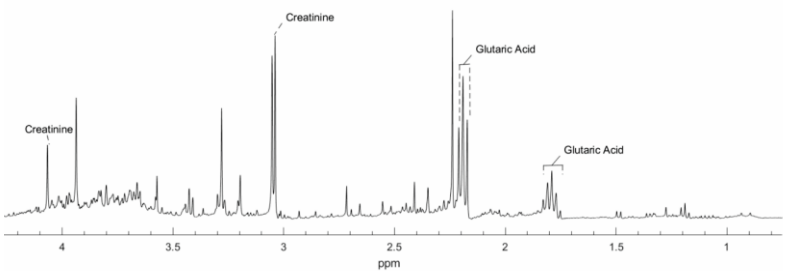

Figure S7. *Glutaric Acidemia Type I:* ^{1}H-NMR spectrum. Signals for Glutaric acid.

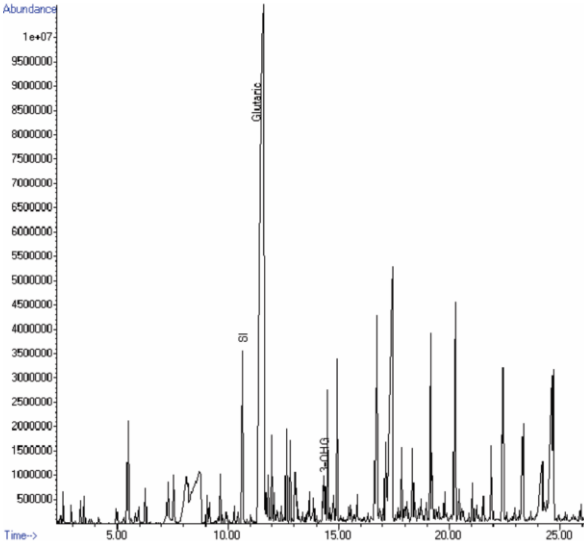

Figure S8. *Glutaric Acidemia Type I:* Chromatogram obtained by GC-MS. Signals for Glutaric acid and 3-Hydroxy-glutaric acid (3-OHG).

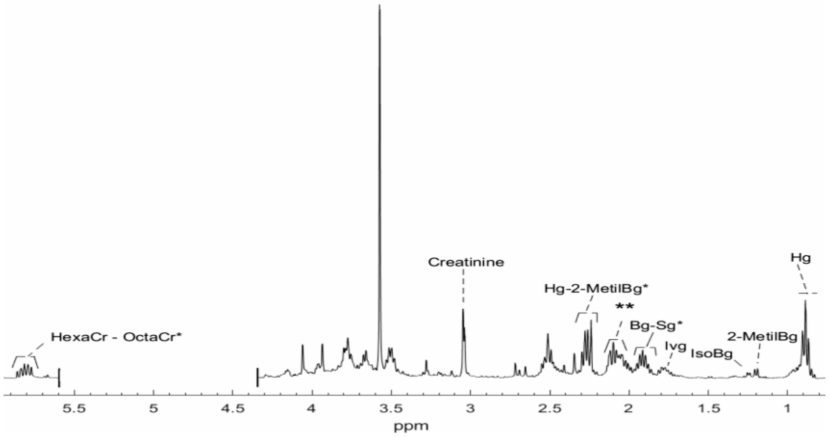

Figure S9. *Medium Chain Acyl-CoA Dehydrogenase Deficiency:* ^{1}H-NMR spectrum. Signals for hexanoylglycine (Hg), 2-methylbutyrylglycine (2-MetilBg), isobutyrylglycine (IsoBg), isovalerylglycine (Ivg), butyrylglycine (Bg) and suberylglycine (Sg), hexanoylcarnitine (HexaCr) and octanoylcarnitine (OctaCr).

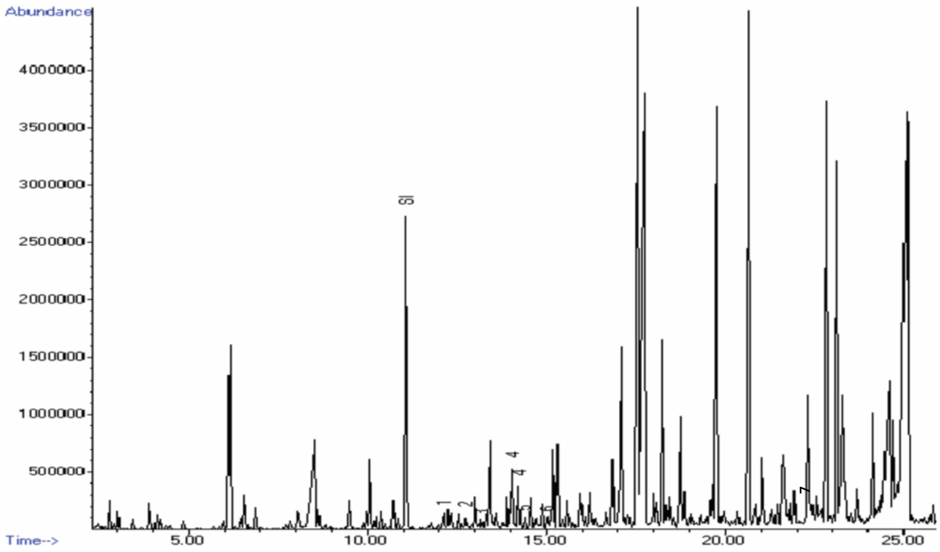

Figure S10 *Medium Chain Acyl-CoA Dehydrogenase Deficiency:* Chromatogram obtained by GC-MS. Signals for **1.**Isobutyrylglycine, **2.**Isovalerylglycine, **3.**Butyrylglycine and 2-Methylbutyrylglycine, **4.**Ty

glylglycine, **5, 6.** Hexanoylglycine**, 7.** Octanoylglycine.

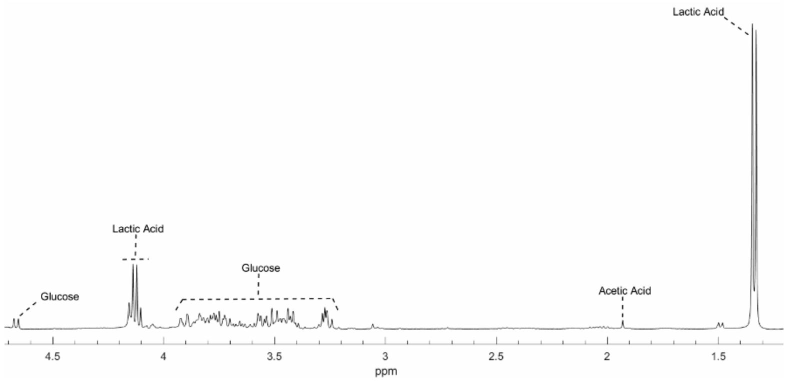

Figure S11. *Lactic Aciduria Condition.* ^{1}H-NMR spectrum. Signals for Lactic Acid, acetic acid and glucose.

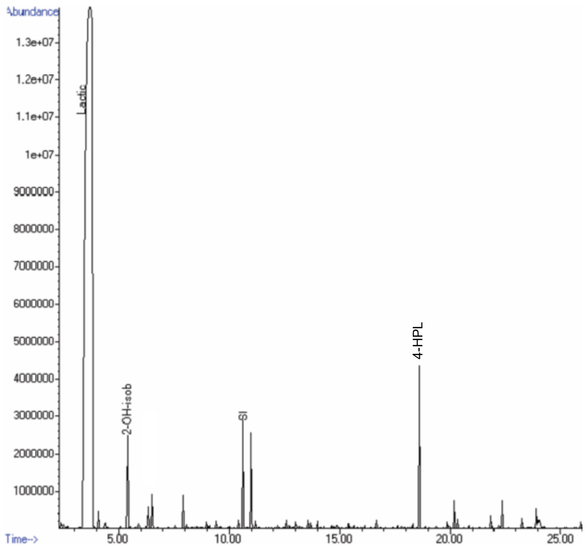

Figure S12. *Lactic Aciduria Condition.* Chromatogram obtained by GC-MS. Signals for Lactic acid, 2-Hydroxy-isobutyric acid (2-OH-isob) and 4-hydroxyphenylactic acid (4-HPL).

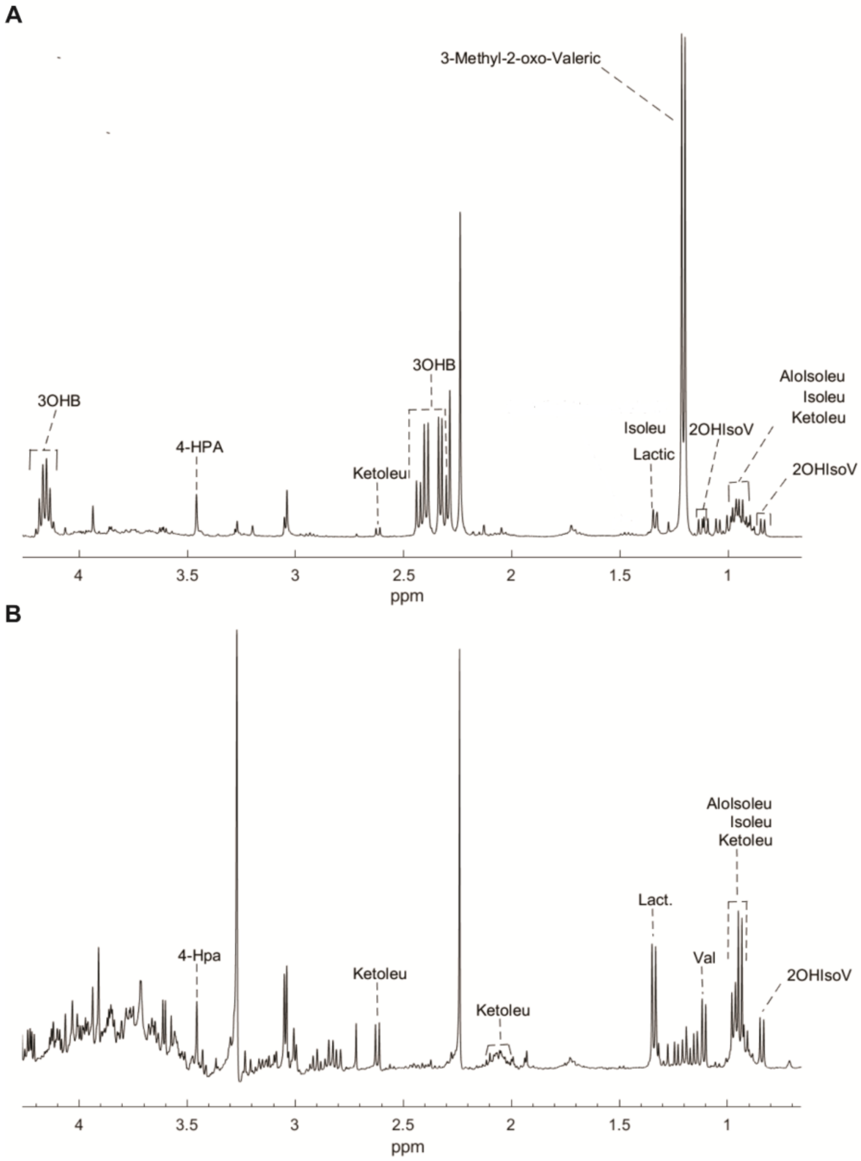

Figure S13. *Maple Syrup Urine Disease.* ^{1}H-NMR spectrum. **(a)** Signals for 2-Hydroxyisovaleric acid (2OHIsoV), D-Alloisoleucine (D-AloIsoleu), D-Isoleucine (D-Isoleu), 2-oxoisocaproic Acid (Ketoleucine), and 3-methyl-2-oxo-valeric acid, 3-Hydroxybutyric acid (3-OHB), **(b)** Signals for Valine (Val), Lactic acid (Lact.) and metabolite of hepatic failure 4-Hydroxy-phenylacetic acid (4-OHPA).

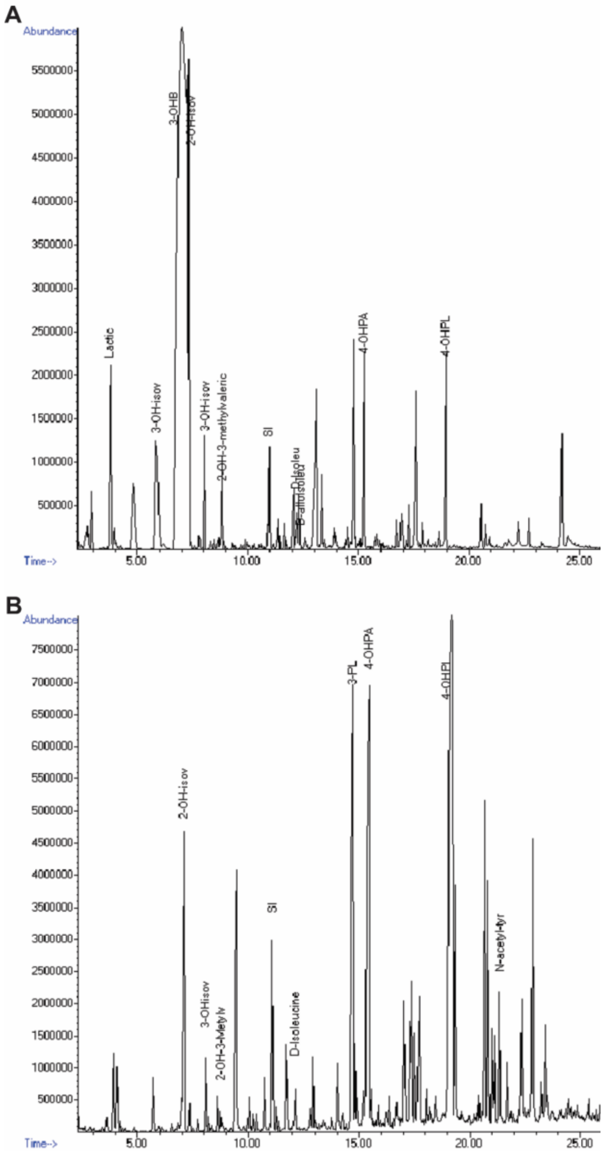

Figure S14. *Maple Syrup Urine Disease.* Chromatograms obtained by GC-MS. **(a)** Signals for Lactic acid, 3-Hydroxybutyric acid (3-OHB), 2-Hydroxyisovaleric acid (2OHIsoV), 2-Hydroxy-3-methyl-valeric acid (2OH-3-Metv), D-Isoleucine (D-Isoleu), D-Alloisoleucine (D-AlloIsoleu). **(b)** Signals for 2-Hydroxyisovaleric acid (2OHIsoV), 3-Hydroxy-isovaleric acid, (3-OH-isov), 2-Hydroxy-3-methyl-valeric acid (2OH-3-Metv), D-Isoleucine, and metabolites of hepatic failure (3-Phenyl-lactic acid (3-PL), 4-Hydroxy-phenylacetic acid (4-OHPA), 4-Hydroxy-phenylactic acid (4-OHPL) and N-acetyl-tyrosine (N-acetyl-tyr).

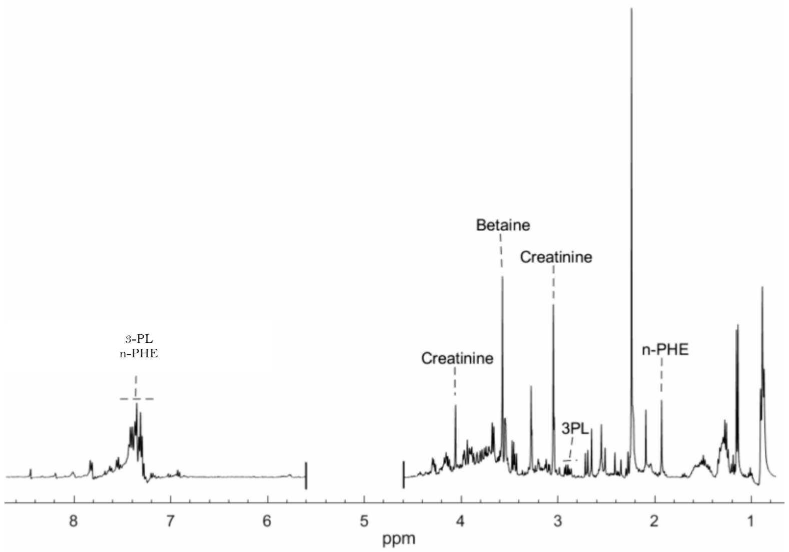

Figure S15. *Phenylalanine metabolism disorders.* ^{1}H-NMR spectrum. Signals for 3-phenyllactic (3-PL) acid and *N*-acetylphenylalanine (n-PHE).

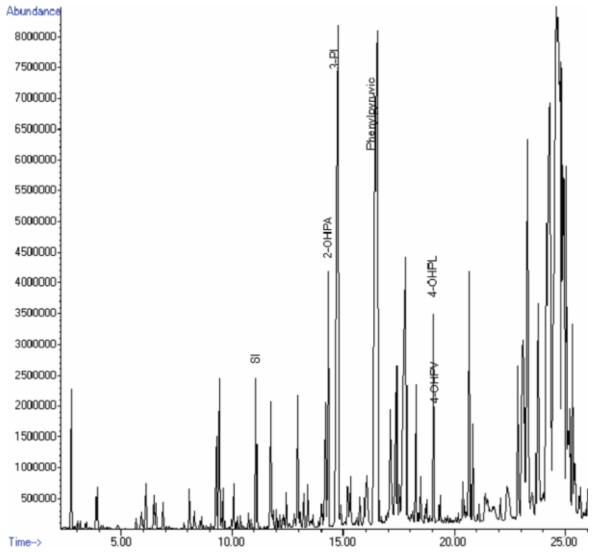

Figure S16. *Phenylalanine metabolism disorders.* Chromatogram obtained by GC-MS. Signals for 2-Hydroxy-phenylacetic acid (2-OHPA), 3-Phenyllactic acid (3-PL), Phenylpyruvic acid, 4-Hydroxy-phenyllactic (4-OHPL) and 4-Hydroxy-phenylpyruvic acid (4-OHPV).

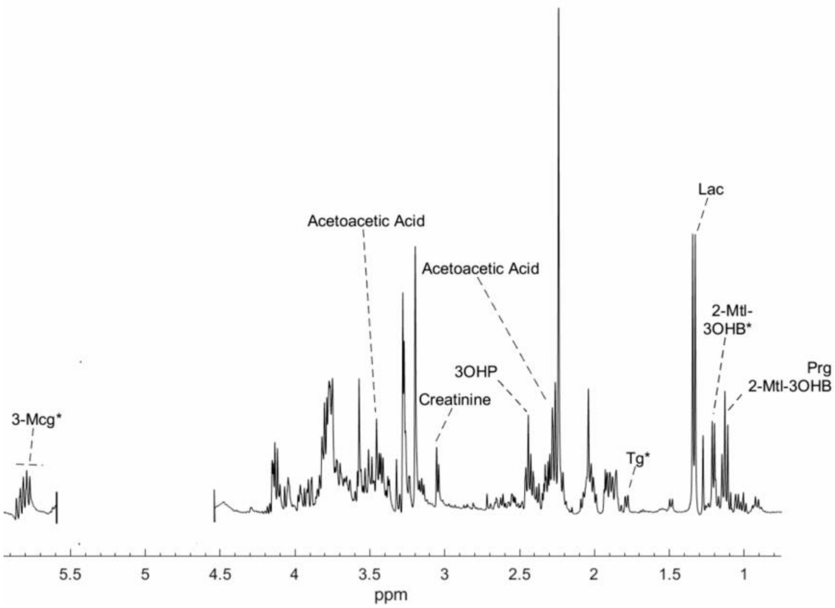

Figure S17. *Holocarboxylase Synthetase Deficiency.* ^{1}H-NMR spectrum. Signals for 2-Methyl-3-hydroxybutyric Acid (2-Mtl-3OHB), Propionylglycine (Prg), Lactic acid (Lac), Tyglylglycine (Tg*), Acetoacetic acid, 3-Hydroxy-propionic acid (3-OHP) and 3-Methylcrotonylglycine.

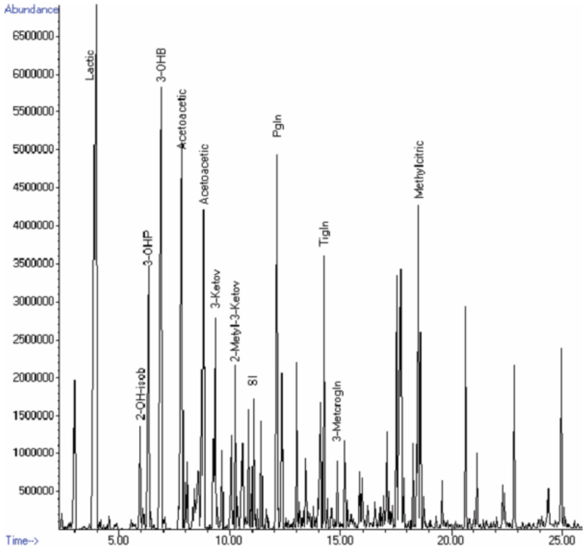

Figure S18. *Holocarboxylase Synthetase Deficiency.* Chromatogram obtained by GC-MS. Signals for Lactic acid, 2-Hydroxy-butyric, 3-Hydroxy-propionic acid (3-OHP), 3-Hydroxybutyric acid (3-OHB), Acetoacetic acid, 3-Ketovaleric acid (3-Ketov), 2-Methyl-3-Ketovaleric acid, Propionylglycine (Pgln), Tyglylglycine (Tigln), Methylcitric Acid, and 3-Methylcrotonylglycine.
